# A Comparison of Nutritional Status, Knowledge and Type 2 Diabetes Risk Among Malaysian Young Adults With and Without Family History of Diabetes

**DOI:** 10.21315/mjms2021.28.1.10

**Published:** 2021-02-24

**Authors:** Farah Yasmin Hasbullah, Kim Yen Fong, Amin Ismail, Joanna Mitri, Barakatun Nisak Mohd Yusof

**Affiliations:** 1 Department of Nutrition and Dietetics, Faculty of Medicine and Health Sciences, Universiti Putra Malaysia, Selangor, Malaysia; 2 Research Centre of Excellence for Nutrition and Non-Communicable Diseases, Faculty of Medicine and Health Sciences, Universiti Putra Malaysia, Selangor, Malaysia; 3 Joslin Diabetes Center, Harvard Medical School, Boston, Massachusetts, United States; 4 Institute for Social Science Studies, Universiti Putra Malaysia, Selangor, Malaysia

**Keywords:** family history, type 2 diabetes, young adults, nutritional status, Malaysia

## Abstract

**Background:**

Genetic factors increase the risk of type 2 diabetes mellitus (T2DM). Thus, family history status may be a useful public health tool for disease prevention. This study compared the nutritional status, knowledge level, and T2DM risk among young adults with and without a family history of diabetes in Malaysia.

**Methods:**

A total of 288 university students aged 18 to 29 years participated in this comparative cross-sectional study. We assessed dietary intake, level of physical activity, knowledge of diabetes and T2DM risk.

**Results:**

Respondents with a family history of diabetes had significantly higher weight (*P* = 0.003), body mass index (*P* < 0.001), waist circumference (*P* < 0.001), diabetes knowledge level (*P* < 0.005) and T2DM risk (*P* < 0.001). Ethnicity, fibre intake, T2DM risk score and knowledge about diabetes were significant contributors toward family history of diabetes (*P* = 0.025, 0.034, < 0.001 and 0.004, respectively).

**Conclusion:**

Young adults with a family history of diabetes had suboptimal nutritional status. Despite being more knowledgeable about diabetes, they did not practice a healthy lifestyle. Family history status can be used to screen young adults at the risk of developing T2DM for primary disease prevention.

## Introduction

Type 2 diabetes mellitus (T2DM) is a major health concern worldwide as it is associated with substantial morbidity and premature mortality and presents several challenges to public health professionals. An estimated 463 million adults are currently living with diabetes worldwide (1) and the figure may rise to 629 million by the year 2045, with a dramatic rise observed in Asia, the epicentre of diabetes (2). According to the global estimates of diabetes prevalence for the year 2013, Asians constitute more than 60% of the world’s population with diabetes (3).

An increasing prevalence of diabetes has been observed among young adults, especially in Asian countries where T2DM develops at a younger age in comparison to their Caucasian counterparts (4). In Malaysia, the T2DM prevalence increased from 15.2% in 2011 to 17.5% in 2015 among adults 18 years and older (5). About 45.8% of diabetes cases in adults were estimated to be undiagnosed and thus might be unaware of their condition (6). Therefore, it is crucial from a clinical and public health perspective to identify high-risk groups.

Multifactorial causes are associated with T2DM, with family health history serving as a critical risk factor that represents genetic information and the complex interplay between shared environment and behavioural effects (7). Having a family history of diabetes is associated with metabolic abnormalities (8), suboptimal nutritional status (9) and an increased risk for future T2DM (10). Although a family history of diabetes is non-modifiable, it may serve as a useful public health tool for disease prevention. Huge biochemical or physical assessments pose a challenge for public health initiatives due to the abundance of patients in the government primary health care clinics (11).

Previous studies have demonstrated that individuals with a family history of diabetes were more knowledgeable about the disease than those without a family history of diabetes (12). However, knowledge was not always associated with a better lifestyle. For example, in the United Kingdom, individuals with a family history of T2DM consumed diets that were predicted to promote rather than prevent T2DM development, despite being more knowledgeable about diabetes (13).

Limited studies have compared the characteristics of young adults with and without a family history of diabetes, particularly in the Asian population (9, 12–15). Moreover, their findings were inconsistent. Young adults had a higher prevalence of a family history of diabetes and elevated body mass index (BMI), and levels of HbA1c and glucose (9, 14–15). Tam et al. (12) found that young adults with a family history of diabetes were significantly more likely to practice a healthy diet. However, they still had a significantly lower physical activity level as compared with those without a family history of diabetes. Moreover, Moon et al. (9) found that calorie intake and regular exercise did not significantly differ according to a family history of diabetes.

Therefore, more research is required to ascertain the findings, particularly among young adults, with ages ranging from 18 to 29 years (16). The presence of obesity and unhealthy lifestyle habits at this life stage is associated with an increased risk of T2DM (17). Understanding the family history status of T2DM among young adults could serve as the basis for delaying or preventing T2DM. Hence, this study compared the nutritional status, knowledge, and T2DM risk among young adults with and without a family history of diabetes in Malaysia.

## Methods

### Study Population

This comparative cross-sectional study compared the nutritional status, knowledge level, and T2DM risk among young adults with and without a family history of diabetes. The study was conducted among undergraduate students aged 18 to 29 years old at Universiti Putra Malaysia (UPM), Serdang, Malaysia. We excluded pregnant or breastfeeding women, individuals who had a confirmed diagnosis of diabetes or those who were uncertain of their family history of diabetes.

Using a cluster sampling method, the recruitment process started at the faculty level. A total of 16 faculties at UPM were stratified into the science, art and technical streams. One faculty was randomly selected from each stream. For each selected faculty, one undergraduate programme was randomly selected. All students in the selected program were then invited to participate in this study. Bachelor of Science (Human Resource and Development) represented the art stream; Bachelor of Engineering (Computer and Communication System Engineering) represented the technology stream and Bachelor of Science (Nutrition and Community Health) represented the science stream.

### Sample Size Calculation

The sample size was calculated using the mean difference formula (18) based on the knowledge of diabetes mellitus (12). A total of 76 respondents per group was sufficient to detect a mean score difference of 0.96 in the diabetes knowledge test between those with and without a family history of diabetes. An additional 20% was required to account for non-response, refusal to participate, or missing data, yielding 95 respondents in each group or 190 total respondents. Next, the sample size (*n* = 190) was multiplied with a design effect of 1.5. Hence, a minimum of 285 respondents were required for the study.

### Measurements

Respondents were asked whether any of their first- or second-degree family members had ‘diagnosed diabetes,’ defined as self-reported doctor-diagnosed diabetes (19). We interviewed the participants for their socio-demographic data, measured the height and weight to derive BMI and waist circumference. BMI was then classified based on the International Obesity Task Force cut-off values for Asian adults (20). The cut-off points of waist circumference (as a measure of central obesity) are ≥ 80 cm and ≥ 90 cm for females and males, respectively (21).

### Dietary Intake

A semi-quantitative food frequency questionnaire (FFQ) was adapted from the Malaysian Adult Nutrition Survey (MANS) (22) to assess dietary intake. For each food item on the list, respondents were asked about the frequency of intake during the last month. They were also asked about the number of servings each time the food was consumed. Each food item listed was given a standard household serving size, which was measured as cooked food or foods ready for consumption (22). The amount of food intake was calculated from food frequency using the following formula (23):

Amount of food (g) per day=frequency of intake (the conversion factor) serving size×total number of servings×weight of food in one serving

Underreporting of energy intake was determined by calculating the ratio between reported total energy intake and basal metabolic rate (EI:BMR), based on the Goldberg cut-off (24). The BMR of the respondents was calculated using the BMR-predictive equation developed by Ismail et al. (25) for Malaysian adults aged 18 to 30 years old.

### Physical Activity Level

The International Physical Activity Questionnaire-Short Form (IPAQ-SF) was used to assess the frequency and duration of physical activity for the last 7 days. IPAQ is reliable and valid in 12 different countries (26), and pre-validated in the previous 2011 Malaysian National Health and Morbidity Survey (NHMS) (27). The volume of activity was calculated by weighting each activity by its energy requirements, defined in METs (metabolic equivalents, which are multiples of the resting metabolic rate) to produce a physical activity score in MET-min (5). Total physical activity scores were obtained by summing up the duration (in min) and frequency (days) of walking, moderate-intensity activity and vigorous-intensity activity (26).

The level of physical activity of respondents was classified as inactive, minimally active and health-enhancing physical activity (HEPA) active based on 2015 Malaysian NHMS guidelines (5). HEPA active individuals are those who engaged in at ≥ 3 days of vigorous-intensity activities, achieving a minimum of 1,500 MET-min/weeks, or a combination of walking, moderate-intensity, or vigorous-intensity activities achieving at least 3,000 MET-min/week (5).

### Knowledge of Diabetes

The diabetes knowledge test was adapted from the Michigan diabetes knowledge test (MDKT) into the Malaysian version (28) and assessed common diabetes-related knowledge. Each item was a close-ended, multiple-choice question with only one correct answer. One point was given for each correct answer and zero points for each wrong answer. The total score ranged from 0 to 14; a higher score indicated a better level of understanding of the disease. The level of diabetes understanding was divided into three categories based on the patient’s total score: low (< 7 points), moderate (7–10 points) and good (≥ 11 points). The Cronbach’s alpha was 0.702, indicating good internal consistency. The test–retest reliability value was 0.894 (*P* < 0.001) (28). This questionnaire had been used among Malaysian adults to determine whether those with family members with diagnosed T2DM or members of different ethnosocial groups were more knowledgeable about diabetes mellitus (12).

### Type 2 Diabetes Risk Assessment

This study used the Australian type 2 diabetes risk assessment tool (AUSDRISK) to predict the 5-year risk of diabetes based on nine risk factors that were either known or easily self-assessed: age, sex, ethnicity, parental history of diabetes, history of high blood glucose level, use of anti-hypertensive medications, smoking, physical inactivity and waist circumference (29). T2DM risk was classified into three categories based on the AUSDRISK score: low risk (< 6 points), intermediate risk (6–11 points) and high risk of developing T2DM (≥ 12 points).

### Statistical Analysis

Data were analysed using IBM SPSS for Windows version 22.0 (SPSS Inc, Chicago, IL, USA). Descriptive data were presented as frequency and percentage for categorical variables, as well as the mean and standard deviation for continuous variables. For between-group comparisons, an independent *t*-test was used to compare continuous variables, whereas Pearson’s χ^2^ test was used to compare categorical variables. We performed binary logistic regression to investigate the contributors to the family history of diabetes. Variables that showed a significant association with T2DM risk in bivariate analysis (*P* < 0.2) were entered in a forward selection multiple regression model. Data were removed if multicollinearity was detected by the variance inflation factor (VIF) ≥ 10 (30). The statistical level of *P* < 0.05 was considered significant.

## Results

**Figure 1:**
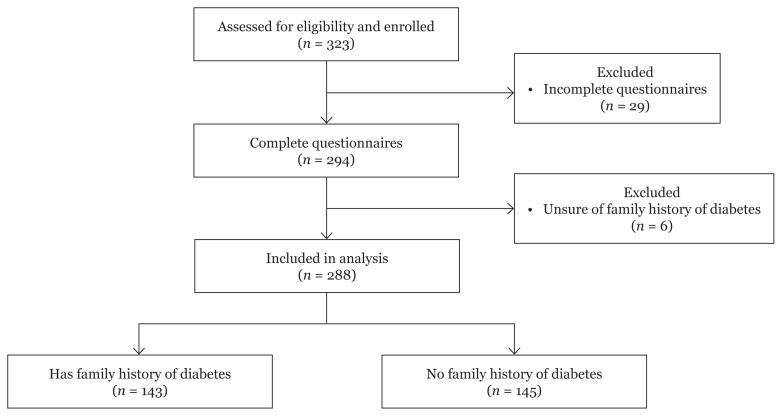
Screening and recruitment of subjects

A total of 323 students from three undergraduate programmes agreed to participate in this study. Out of these, 294 (91.0%) completed the questionnaires. Six respondents were excluded as they did not know their family history of T2DM. Hence, 288 young adults with and without a family history of T2DM were included in the final analysis. The mean age was 21.7 years (SD = 1.5) (Figure 1).

The respondents were predominantly female (78.1%) of Malay ethnicity (73.3%) and living on campus (92.7%). More than half of the respondents were from the Nutrition and Community Health programme (52.7%), and the highest proportion of the respondents constituted first-year students (31.9%). There were no differences in socio-demographic characteristics between the two groups (Table 1).

For respondents with a family history of T2DM, the majority of them had parents diagnosed with T2DM (46.2%), followed by paternal/maternal grandparents (36.4%), and paternal/maternal uncles or aunts (16.1%). Only 1.4% of respondents had siblings diagnosed with T2DM.

Compared with the other group, respondents with a family history of T2DM had significantly more weight (*P* = 0.003), higher BMI (*P* < 0.001) and larger waist circumference (*P* < 0.001). Moreover, a significantly higher proportion of respondents with a family history of T2DM was overweight (*P* = 0.029) and had waist circumference above the recommended range (*P* = 0.019) (Table 2).

The two groups had comparable intakes of energy and macronutrients. Mean carbohydrate, protein and fat intakes were also within the recommended range in both groups. The two groups did not underreport their dietary intake, as shown by the mean EI:BMR ratio of > 1.48. Furthermore, the two groups did not differ in terms of total physical activity score. However, fewer respondents with a family history of T2DM were HEPA active than those without a family history of T2DM (20.0% versus 46.2%; *P* = 0.012) (Table 2).

Respondents with a family history of T2DM scored significantly higher on the diabetes knowledge test (*P* = 0.009). A significant proportion of them had a high knowledge level (*P* = 0.002) and scored correctly on Question 3 regarding high-fat foods (*P* = 0.012). Responses to other questions on the diabetes knowledge test were comparable between the two groups. However, questions with the lowest correct responses (< 40%) were on diet (Questions 4 and 7) (Table 3).

Respondents with a family history of T2DM had a significantly higher risk of T2DM (*P* < 0.001). A significantly higher proportion of respondents with a family history of T2DM also had an intermediate risk (39.9% versus 17.9%) and high risk (8.4% versus 1.4%) (*P* < 0.001) (Table 3).

Logistic regression analysis revealed that significant contributors to family history of diabetes were non-Malay ethnicity (adjusted odds ratio [AOR] 2.048; *P* = 0.025); fibre intake (g/1,000 kcal) (AOR 1.272; *P* = 0.034), T2DM risk score (AOR 1.329; *P* < 0.001) and diabetes knowledge score (AOR 1.2; *P* = 0.004). The model contributed to 21.5% of variations in the family history of diabetes (*P* < 0.001) (Table 4).

## Discussion

This study compared the nutritional status, knowledge and risk of T2DM scores among young adults with and without a family history of T2DM. Respondents with a family history of T2DM had significantly increased weight, BMI and waist circumference. The finding was in line with a previous cross-sectional study conducted among young adults in Italy, in which individuals with a first- or second-degree family history of diabetes had significantly increased BMI (*P* < 0.001 for both sexes), weight and waist circumference (*P* < 0.005 for men, *P* < 0.0001 for women) compared with those without a family history of diabetes (31).

Obesity is an established risk factor of T2DM, accentuated by the presence of a family history of diabetes (31). A family history of diabetes was associated with both increased obesity risk and susceptibility to the negative effects of excess body fat (32). T2DM risk is associated with incremental increases in body weight in young adulthood, especially for those with a family history of diabetes (33). In addition, increased waist circumference may indicate intra-abdominal obesity, which is associated with insulin resistance, thus increasing the likelihood of developing T2DM (34). Abdominal obesity may cause fat cells to release pro-inflammatory chemicals, leading to insulin insensitivity (35).

Our study suggests that a family history of diabetes or genetic predisposition results in different body compositions in young adults. These results increase the concern for developing diabetes as Asians are reported to have a high proportion of body fat and prominent abdominal obesity as compared to their Caucasian populations, even at similar BMI values (36). Thus, Asians are more predisposed to insulin resistance and develop diabetes at a lower degree of obesity (36). Furthermore, Asians were reported to develop early β-cell failure (37), thereby lies the importance of identifying young adults with a family history of diabetes and subsequent intervention.

Respondents with a family history of T2DM were significantly more knowledgeable about diabetes in this study. Our results aligned with another study conducted among healthy Malaysian individuals (mean age: 30.97 years old), in which those with a family history of diabetes were significantly more knowledgeable about diabetes (*P <* 0.001) (12). However, it is intriguing that these more knowledgeable individuals were less physically active (*P* < 0.01). The findings suggested that knowledge was not translated into a more optimal and healthier lifestyle.

In our study, questions with the lowest correct responses among all respondents (< 40%) were on diet (Questions 4 and 7). Despite having good knowledge about the basic concepts of diabetes, most of them had low knowledge of the relationship between dietary intake and blood glucose. Thus, effective prevention strategies should incorporate nutrition knowledge for young adults to adopt a healthier diet that could reduce their T2DM risk.

We observed that young adults with a family history of T2DM had a significantly higher score of T2DM risks (*P* < 0.001). Our findings supported those from the Korea National Health and Nutrition Survey (9), in which adults aged 25 to 44 years old with a first-degree family history of diabetes had a significantly higher prevalence of T2DM compared with those without a family history of diabetes. A family history of diabetes increased the incidence of T2DM by 1.4- to 6.1-fold (38). The manifestations of genetic susceptibility included reduced insulin secretion and insulin insensitivity, even in otherwise healthy young adults (39). Coupled with environmental factors including obesity and sedentary lifestyle, genetic susceptibility may ultimately translate to T2DM (39). Thus, family history should be considered as an inexpensive and promising health tool to estimate metabolic outcomes such as T2DM (9).

The complicated relationship between diet and disease cannot be understood by only studying a single dietary component (40). The use of dietary patterns can better predict the relationship between diet and T2DM prevention. Dietary patterns characterised by high intakes of sugar-sweetened beverages, processed meat and refined grains are independently associated with the risk of T2DM in multiple cohorts (41). However, our findings also suggest a crucial function of genetics in disease risk, which should be factored in when planning dietary interventions. We postulate that dietary intervention strategies to prevent T2DM among those with a family history of diabetes could differ from those without a family history.

To our knowledge, this is the first study that compared the risk of T2DM among Malaysian young adults with and without a family history of diabetes from several ethnicities. However, the study has certain limitations. It was a cross-sectional design, which did not allow a causal relationship between family history and T2DM risk.

We also did not assess the biochemical profile of the respondents. In the Korea National Health and Nutrition Survey, adults with a family history of diabetes had significantly increased levels of fasting glucose and triglycerides, and lower β-cell function despite having an optimal glucose tolerance status (9). Thus, young adults with a family history of diabetes in our study could already be having metabolic syndrome, predisposing them to the risk of T2DM, despite being more knowledgeable and having similar dietary intake as those without a family history of diabetes. Weight, waist circumference and BMI were significantly increased in respondents with a family history of diabetes, suggesting that they might have metabolic syndrome, which warrants further investigation. In addition, blood concentrations of several metabolites, including branched-chain amino acids, triglycerides and hexoses, increased in individuals with pre-diabetes and T2DM (42). Hence, future studies should assess both dietary patterns and their metabolomic markers to obtain a better understanding of the relationship between family history of diabetes, diet and T2DM risk.

## Conclusion

Young adults with a family history of diabetes had suboptimal nutritional status and higher T2DM risk as compared with those without a family history of diabetes. Although they were more knowledgeable about diabetes, they did not follow a healthy diet and lifestyle. Family history of diabetes could be used as a screening tool to identify young adults at high and moderate risk of developing T2DM. These individuals may benefit from targeted lifestyle intervention to delay the development of diabetes through weight loss and increasing physical activity. Hence, young adults must keep a record of their family medical history, which could facilitate the evaluation of their future risk of developing T2DM. Future studies among young adults are required with an emphasis on their dietary pattern and metabolomic markers to determine strategic interventions to delay or prevent the onset of T2DM.

## Figures and Tables

**Table 1:** Socio-demographic characteristics of respondents with and without a family history of T2DM (*N* = 288)

Variables	Total (*N* = 288) *N* (%)	With a family history of T2DM (*n* = 143) *n* (%)	Without a family history of T2DM(*n* = 145) *n* (%)	*P*-value
Age (years)^a^	21.7 (1.5)	21.7 (1.5)	21.7 (1.5)	0.594
Sex				
Male	63 (21.9)	28 (19.6)	35 (24.1)	0.35
Female	225 (78.1)	115 (80.4)	110 (75.9)	
Ethnicity				
Malay	211 (73.3)	112 (78.3)	99 (68.3)	0.081
Chinese	53 (18.4)	20 (14.0)	33 (22.8)	
Indian	11 (3.8)	7 (4.9)	4 (2.8)	
Others	13 (4.5)	4 (2.8)	9 (6.2)	
Academic programme				
Nutrition and Community Health	155 (53.8)	82 (57.3)	73 (50.3)	0.464
Computer and Communication System Engineering	72 (26.0)	34 (23.8)	38 (26.2)	
Human Resource Development	61 (21.2)	27 (18.9)	34 (23.5)	
Year of study				
First year	92 (31.9)	49 (34.3)	43 (29.7)	0.266
Second year	86 (29.9)	35 (24.5)	51 (35.2)	
Third year	51 (17.7)	27 (18.9)	24 (16.6)	
Final/Fourth year	59 (20.5)	32 (22.4)	27 (18.6)	
Living arrangement				
College dormitory	267 (92.7)	133 (93.0)	134 (92.4)	0.846
Rented room/own house	21 (7.3)	10 (7.0)	11 (7.6)	
Father’s education level				
Primary school	22 (7.6)	7 (4.9)	15 (10.3)	0.218
Secondary school	181 (62.8)	93 (65.1)	88 (60.7)	
College/university	85 (29.5)	43 (30.1)	42 (29.0)	
Mother’s education level				
Primary school	29 (10.1)	10 (7.0)	29 (20.0)	0.197
Secondary school	203 (70.5)	106 (74.2)	97 (66.9)	
College/university	56 (19.4)	27 (18.9)	19 (13.1)	
Household income				
≤ RM1,000	43 (14.9)	21 (14.7)	22 (15.2)	0.322
RM1,001–RM2,300	84 (29.2)	44 (30.8)	40 (27.6)	
RM2,301–RM5,599	100 (34.7)	43 (30.1)	57 (39.3)	
≥ RM5,600	61 (21.2)	35 (24.5)	26 (17.9)	
Financial support				
Self-funded	54 (18.8)	26 (18.2)	28 (19.3)	0.927
Scholarship	58 (20.1)	30 (21.0)	28 (19.3)	
Study loan	176 (61.1)	87 (60.8)	89 (61.4)	

Notes:

a mean (SD), tested using independent *t*-test; Others: Pearson’s χ^2^ test

**Table 2:** Nutritional status of respondents with and without a family history of T2DM (*N* = 288)

Variables	With a family history of T2DM (*n* = 143)	Without a family history of T2DM (*n* = 145)	*P*-value

	mean (SD)^a^		
Weight (kg)	58.3 (12.48)	54.3 (10.30)	0.003
Height (cm)	158.8 (7.67)	158.9 (8.72)	0.918
BMI (kg/m^2^)	23.10 (4.65)	21.51 (3.46)	< 0.001
Waist circumference (cm)	72.65 (9.08)	69.18 (7.94)	< 0.001
Energy intake (kcal/day)	2,471 (1,056)	2,505 (980)	0.779
EI:BMR ratio	2.0 (0.9)	2.1 (0.9)	0.949
Carbohydrate intake			
Total (g/day)	314.8 (126.3)	321.0 (127.0)	0.932
% from energy intake	51.8 (6.3)	51.6 (6.8)	0.109
Protein intake			
Total (g/day)	92.9 (51.9)	92.4 (37.5)	0.198
% from energy intake	15.0 (3.5)	14.9 (2.8)	0.741
Fat intake			
Total (g/day)	85.8 (49.0)	86.0 (40.0)	0.960
% from energy intake	30.6 (5.2)	30.5 (5.6)	0.247
Fibre			
g/day	6.4 (4.1)	6.2 (3.6)	0.554
g/1000 kcal	2.67 (1.60)	2.41 (1.00)	0.097
Physical activity (MET-minute/week)			
Total	2,854.81 (2,849.57)	1,927.61 (2,148.73)	0.487
Walking (3.3 METs)	1,904.77 (2,298.16)	1,927.61 (2,148.73)	0.931
Moderate intensity (4.0 METs)	344.84 (529.53)	455.59 (764.35)	0.155
Vigorous intensity (8.0 METs)	605.20 (1,078.26)	715.03 (1,379.20)	0.453

	***n* (%)**^b^		

BMI classes			
Underweight (< 18.5)	15 (10.5)	23 (16.6)	
Normal (18.5–22.99)	70 (49.0)	84 (57.2)	0.029
Overweight (≥ 23.0)	58 (40.6)	38 (26.2)	
Waist circumference			
Above recommendation^c^	21 (14.7)	9 (6.2)	0.019
Within receomendation^d^	122 (85.3)	136 (93.8)	
Physical activity category			
Inactive	40 (28.0)	49 (33.8)	
Minimally active	51 (35.7)	29 (20.0)	0.012
cHEPA active	52 (36.4)	67 (46.2)	

Notes:

a Tested using independent *t*-test;

b Tested using Pearson’s χ^2^ test;

c Waist circumference ≥ 80 cm for females or ≥ 90 cm for males;

d Waist circumference < 80 cm for females or < 90 cm for males

**Table 3:** Knowledge of diabetes and T2DM risk of respondents with and without a family history of T2DM (*N* = 288)

Variables	With a family history of T2DM(*n* = 143)	Without a family history of T2DM(*n* = 145)	*P*-value
Knowledge score^a^	7.73 (2.07)	7.06 (2.26)	0.009

	***n* (%)**	***n* (%)**	

Knowledge level			
Low	55 (38.5)	81 (55.9)	0.002
Moderate	81 (56.6)	52 (35.9)	
High	7 (4.9)	12 (8.3)	
Correct responses			
Q1 About diabetes diet	56 (39.2)	45 (31.0)	0.148
Q2 About high carbohydrate foods	117 (81.8)	113 (78.0)	0.411
Q3 About high-fat foods	93 (65.0)	73 (50.3)	0.012
Q4 About tips for choosing safe foods to be taken for people with diabetes	37 (25.9)	37 (25.5)	0.945
Q5 About glycosylated haemoglobin (HbA1c) test	54 (37.8)	49 (33.8)	0.482
Q6 About method of testing blood glucose	88 (61.5)	86 (59.3)	0.699
Q7 About unsweetened fruit juice	41 (28.7)	31 (21.4)	0.153
Q8 About foods used to treat low blood glucose	56 (39.2)	56 (38.6)	0.925
Q9 About effect of exercise on blood glucose	99 (69.2)	101 (69.7)	0.938
Q10 About effect of infections on blood glucose	64 (44.8)	60 (41.4)	0.563
Q11 About ways to take care of the feet for people with diabetes	93 (65.0)	87 (60.0)	0.378
Q12 About effect of eating foods low in fat	116 (81.1)	106 (73.1)	0.106
Q13 About complications related to numbness and tingling	105 (73.4)	93 (64.1)	0.089
Q14 About complications of diabetes	87 (60.8)	87 (60.0)	0.884
T2DM risk assessment score^a^	6.69 (3.23)	4.94 (2.30)	< 0.001
Low risk (< 6 points)	74 (51.7)	117 (80.7)	
Intermediate risk (6–11 points)	57 (39.9)	26 (17.9)	< 0.001
High risk (≥ 12 points)	12 (8.4)	2 (1.4)	

Notes:

a mean (SD), tested using independent *t*-test; Others: Pearson’s χ^2^ test

**Table 4:** Logistic regression analyses on contributors of family history of diabetes (*N* = 288)

Variables	AOR	95% CI	*P*-value
Ethnicity	2.048	1.096, 3.826	0.025*
Mother’s education level	1.195	0.622, 2.297	0.592
Body mass index (kg/m^2^)	0.994	0.868, 1.137	0.925
Waist circumference (cm)	1.004	0.937, 1.076	0.902
Fibre intake (g/1,000 kcal)	1.272	1.018, 1.589	0.034*
Physical activity level	0.609	0.349, 1.062	0.080
Diabetes knowledge score	1.200	1.170, 1.508	0.004*
T2DM risk score	1.329	1.062, 1.357	< 0.001**

Notes:

* *P* < 0.05;

** *P* < 0.001; adjusted R^2^ = 21.5; model *P* < 0.001
